# Wood-Based Carbon Quantum Dots Regulate Photosynthetic Carbon Fixation and Enhance Mechanical Properties of Soybean Straw

**DOI:** 10.3390/polym18202538

**Published:** 2026-10-16

**Authors:** Yujia Fu, Fengqi Qiu, Sihan Tu, Zhenhua Xue

**Affiliations:** 1 College of Material Science and Art Design, Inner Mongolia Agricultural University, Hohhot 010018, China; 2 Inner Mongolia Key Laboratory for Sand Shrubs Fibrosis and Energy Development and Utilization, Hohhot 010018, China; 3 National Forestry Grassland Engineering Technology Research Center for Efficient Development and Utilization of Sandy Shrubs, Hohhot 010018, China

**Keywords:** carbon quantum dots, biomass materials, cellulose accumulation, photosynthetic carbon fixation, mechanical properties

## Abstract

In order to achieve the goal of ‘double carbon’ and promote the high-value utilization of biomass materials, nitrogen-doped carbon quantum dots (N-CQDs) with high crystallinity were successfully prepared by one-step hydrothermal method using renewable Salix powder as a precursor, and citric acid and urea as an activator and nitrogen source. The research focused on the regulation mechanism of N-CQDs as nano-modulators on photosynthetic carbon fixation of soybean plants, accumulation of micro-structural carbohydrates (cellulose/lignin) in the body, and finally the macro-mechanical strength of soybean straw. The results showed that N-CQDs had a significant ‘low promotion and high inhibition’ effect on the physiological metabolism of soybean. A 5 mg/L low concentration treatment could effectively expand the blue-violet light capture area, accelerate the Hill reaction electron transfer on the thylakoid membrane, and significantly increase the cellulose content in soybean stem by 15.4% compared with the control group. In terms of macroscopic mechanical properties, the shear force of soybean straw in the best treatment group reached 134.76 N, the maximum tensile stress reached 4.25 MPa, and the average increase was 58%. However, the high concentration treatment of 50 mg/L resulted in a sharp decrease of 17.7% in cellulose content and a decrease in tensile stress to 2.21 MPa due to nanotoxicity or light saturation inhibition. In this study, a closed loop of ‘nanodot synthesis-microchemical composition evolution-macromaterial mechanical mapping’ was constructed, which provided a new strategy for source regulation and performance enhancement of biomass functional materials.

## 1. Introduction

With the increasing global warming and carbon emission problems, the conversion of atmospheric CO_2_ into unsaturated organic carbon chains and fixation in plants through plant photosynthesis is recognized as the most economical and environmentally friendly biological carbon sequestration and increases carbon sink pathway [1,2]. However, under natural growth conditions, green plants have a physiological bottleneck due to the narrow light absorption bandwidth of photosynthetic pigments and low enzyme catalytic rate, resulting in an extremely low overall light energy utilization rate (usually less than 10%), which limits the inorganic carbon assimilation and metabolic conversion efficiency of CO_2_ [3,4]. In recent years, the rise in nano-plant biology has provided a new perspective for breaking through the natural physiological limits of plants. Researchers have found that the introduction of engineering nanomaterials with specific optical and electrochemical properties as ‘exogenous regulators’ can effectively enhance the efficiency of chloroplasts in capturing sunlight, energy conversion and transfer, thus efficiently converting solar energy into high-value biomass energy [5,6].

Among many nano-regulation materials, carbon quantum dots (CQDs) have shown great potential in the field of plant physiological regulation due to their unique advantages such as particle size less than 10 nm, excellent water solubility, low cytotoxicity and good biocompatibility [7,8]. Exogenously applied CQDs can penetrate multiple biological barriers and penetrate into plant tissues. Using their unique photoluminescence (PL) and down-conversion characteristics, CQDs can be used as efficient ‘exogenous light-harvesting antenna’ to absorb ultraviolet light that is difficult for plants to use directly and convert in situ to emit strong blue-violet light, which overlaps with the absorption spectrum of chloroplast photosynthetic pigments to form a high spectral line overlap [9,10]. This spectral sensitization mechanism can significantly enhance the light-harvesting capacity of chloroplasts and accelerate the photosynthetic electron transfer rate from photosystem II (PSII) to photosystem I (PSI) on thylakoid membranes during the photoreaction stage, thus providing a larger assimilation force to support the Calvin cycle of downstream dark reactions [11,12].

However, most of the existing frontier research focuses on the apparent improvement of crop yield or stress resistance phenotype by CQDs; there is still a lack of systematic research on how CQDs trigger thylakoid electron transfer from the source and regulate the secondary accumulation of structural carbohydrates (such as cellulose and lignin) in the downstream plant stems, and then how to map and change the complete chain of mechanical properties of the final macroscopic biomass functional materials [13,14]. As a typical green plant biomass material, the physical aggregation and chemical crosslinking of the cellulose skeleton and lignin network in the cell wall of soybean straw and other cell walls have a decisive influence on the subsequent functional high-value utilization and engineering mechanical characterization [15,16].

Based on this, this study used renewable salix powder as a low-cost precursor, introduced citric acid and urea as activators and nitrogen sources, and synthesized nitrogen-doped carbon quantum dots (N-CQDs) with high crystallinity by a one-step hydrothermal method. Taking soybean plants as the research model, the transport pathway of exogenous N-CQDs in plants and the pressurizing mechanism of photosynthetic electron transport chain were systematically deconstructed by Hill reaction kinetics monitoring of in vitro hybrid photosynthetic system and in situ laser confocal fluorescence tracing in vivo. On this basis, the composition evolution of non-structural carbohydrates (soluble sugar) and structural carbohydrates (cellulose/lignin) in dry stems under different regulatory concentrations was quantitatively determined, and the biochemical metabolic boundary of ‘low promotion and high inhibition’ was clarified. Finally, the test system of mechanical shear and tensile properties of straw was constructed, in order to provide a solid theoretical support for the source ecological orientation cultivation and material performance regulation of biomass functional materials.

## 2. Synthesis, Optimization and Characterization of Wood-Based Carbon Quantum Dots

### 2.1. Test Precursor Source and Specification

In this study, natural salix powder was used to prepare carbon quantum dots. The sand willow used was sourced from the school’s factory at Inner Mongolia Agricultural University (Sandy Willow Wood Powder was peeled and ground into 60 mesh wood powder (wood meal)). It was crushed by a high-speed pulverizer and screened by 60 mesh. Other chemical reagents used in the test include lignin (analytically pure, Shanghai McLin Biochemical Technology Co., Ltd., Shanghai, China), urea (analytically pure, Tianjin Xinbot Chemical Co., Ltd., Tianjin, China) and citric acid (analytically pure, Tianjin Xinbot Chemical Co., Ltd.). The preparation method is as follows:

CQDs were synthesized by hydrothermal method using Salix powder as precursor. The Salix wood was peeled and ground into 60 mesh wood powder, and 1.1 g of Salix wood powder was dissolved in 30 mL of deionized water and dispersed by ultrasonic for 10 min. After ultrasonication, the mixture was transferred to a stainless-steel autoclave lined with 100 mL polytetrafluoroethylene and reacted at 180 °C for 12 h. The reactant was initially filtered by centrifugation at 8000 rmp for 10 min, and then filtered through a 0.22 μm filter membrane to remove the large aggregates of wood-based CQDs. Finally, the yellowish-brown liquid obtained by filtration was dialyzed and purified in a 1000 Da molecular weight cut-off dialysis bag in deionized water for 48 h. After freeze-drying, biomass carbon quantum dots were obtained and stored in dark to obtain CQDs.

**Figure 1:**
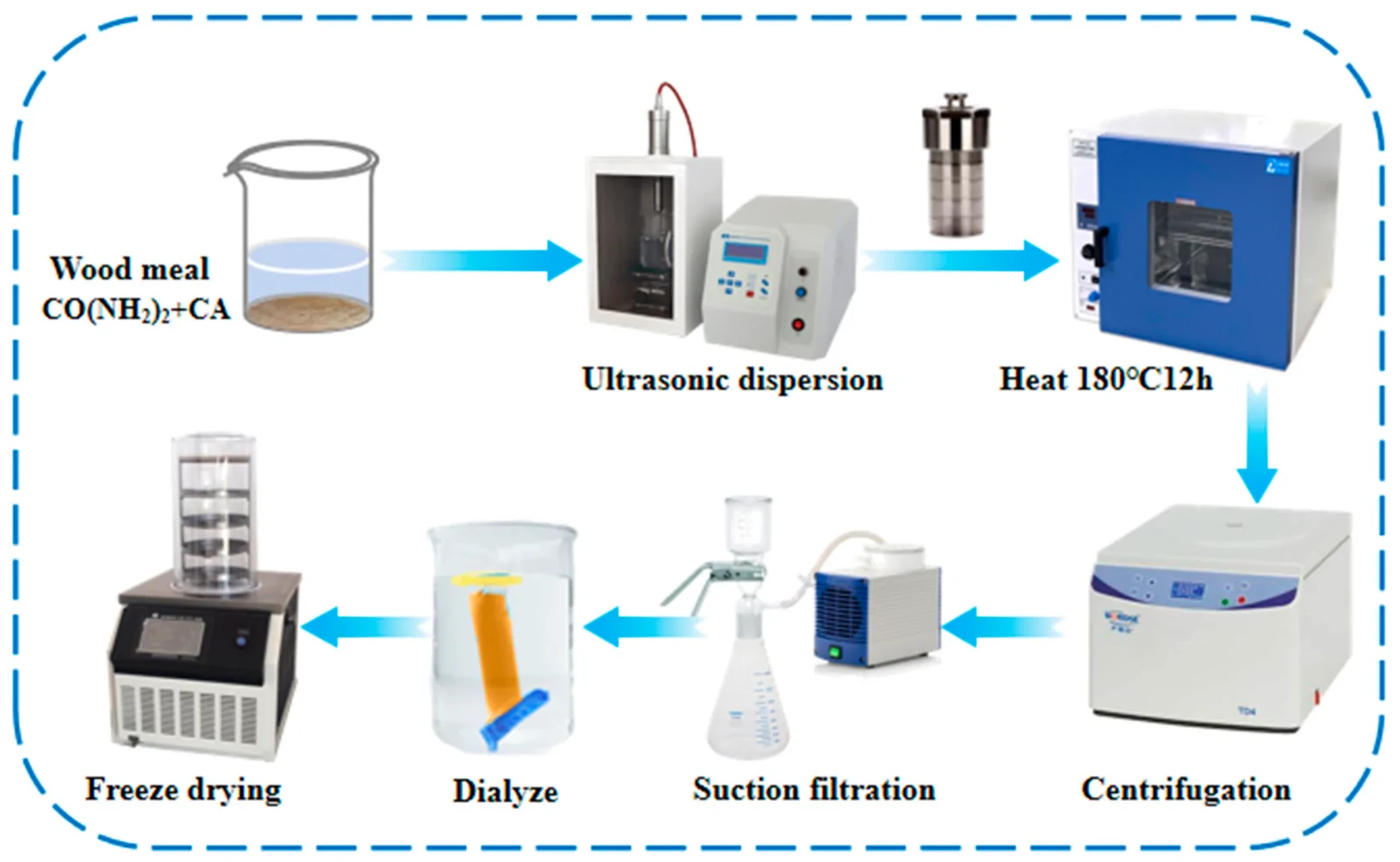
Preparation flow chart of CQDs and N-CQDs (where “wood meal” denotes finely pulverized Salix wood powder sieved through 60-mesh).

N-CQDs were synthesized by one-step hydrothermal method using Salix powder as carbon source, urea as nitrogen source, and citric acid as carbon source to increase its surface functional groups. The amount of Salix powder added was 1.1 g, citric acid was 1 g, and urea was 2 g, which were dissolved in 30 mL deionized water in turn. The preparation process was the same as above. N-CQDs were obtained after ultrasonic, heating, filtration, dialysis and freeze-drying, the detailed preparation process is shown in Figure 1.

### 2.2. Characterization of Structure and Morphology of Quantum Dots

#### 2.2.1. Analysis of Surface Chemical Composition and Heteroatom Cross-Linking Configuration

**Figure 2:**
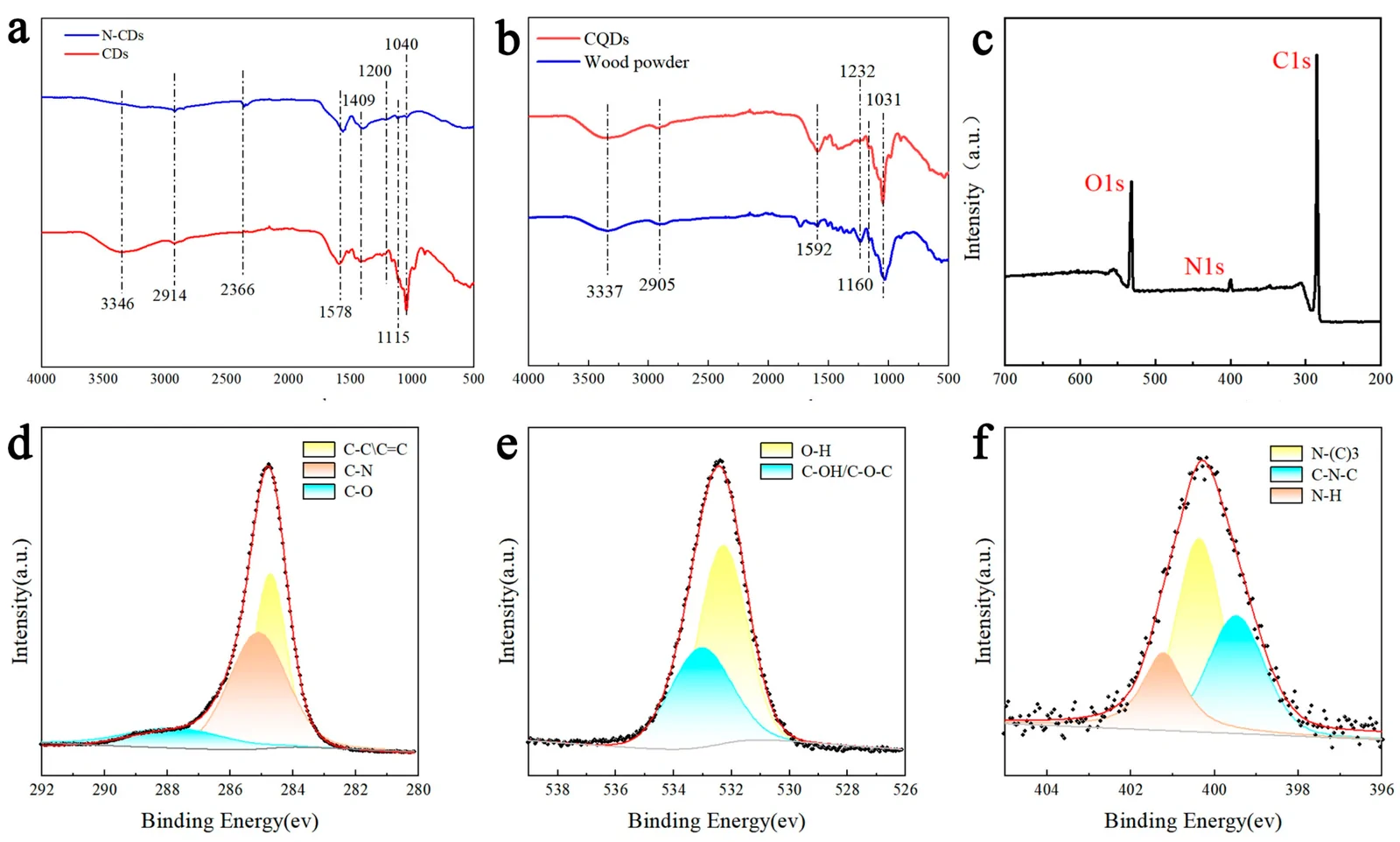
(**a**,**b**) Infrared spectra of different materials; XPS spectra of N-CQDs ((**c**): XPS total spectra; (**d**): C1s fine spectra; (**e**): O1s fine spectrum; (**f**): N1s fine spectra).

The chemical composition of CQDs and N-CQDs was studied by Fourier transform infrared (FT-IR) spectroscopy, and the binding mode of N-doped CQDs was further investigated. The results are shown in Figure 2. The absorption peaks at 3346 cm^−1^, 2914 cm^−1^ and 1578 cm^−1^ of the prepared CQDs and urea-doped N-CQDs are due to the stretching vibration of the O–H bond, the stretching vibration of the C–H bond, and the stretching vibration of the C=C bond, respectively. The peaks at 1200 cm^−1^ and 1115 cm^−1^ are C–O and C–O–C stretching vibrations [17]. The C–N stretching vibration absorption peak of urea-doped carbon quantum dots appeared at 1409 cm^−1^, indicating that nitrogen element was successfully doped [18].

The characteristic peaks of Salix powder and its prepared CQDs-1 are basically the same as shown in Figure 2b, indicating that the main skeleton structure of Salix powder is still retained. The peaks at 1232 cm^−1^ and 1160 cm^−1^ correspond to C–O and C–O–C stretching vibrations, respectively. The absorption peaks at 3337 cm^−1^, 2905 cm^−1^ and 1592 cm^−1^ are due to the stretching vibration of the O–H bond, the stretching vibration of the C–H bond and the stretching vibration of the C=C bond, respectively. The stretching vibration of the C=C bond indicates that there are many conjugated groups, which is one of the reasons for the luminescence of CQDs.

The N-CQDs were characterized by XPS, and the chemical composition and chemical structure of the samples were analyzed. As shown in Figure 2c, there are obvious peaks at 285, 400 and 532 eV, corresponding to C1s, N1s and O1s, respectively. As shown in the C1s spectrum (Figure 2d), the peaks at 284.7, 285.1 and 287.7 eV are C–C\C=C, C–N and C–O bonds, respectively. The O1s spectrum of N-CQDs (Figure 2e) shows two peaks at 533.0 eV and 532.3 eV, which are C–OH/C–O–C and O–H bonds, respectively. In addition, the N1s spectra of N-CQDs (Figure 2f) were fitted to three N-related bonds of C–N–C, N–H and N–(C)_3_ at 399.3, 401.2 and 400.4 eV, respectively. It can be seen that the prepared N-CQDs are nitrogen-doped and carbon-rich polymer nanodots, and their fluorescence intensity increases due to the presence of substituents such as O–H, N–H, and C–N–C. In addition, the C–N bond appearing at 1409 cm^−1^ in the FTIR spectrum also confirmed this. Due to the doping of the N element and the presence of substituents such as O–H, N–H, and C–N–C, the electron delocalization ability of N-CQDs is enhanced, and its luminescence intensity and water solubility are significantly optimized [19,20].

#### 2.2.2. Microscopic Morphology and Lattice Diffraction Analysis

**Figure 3:**
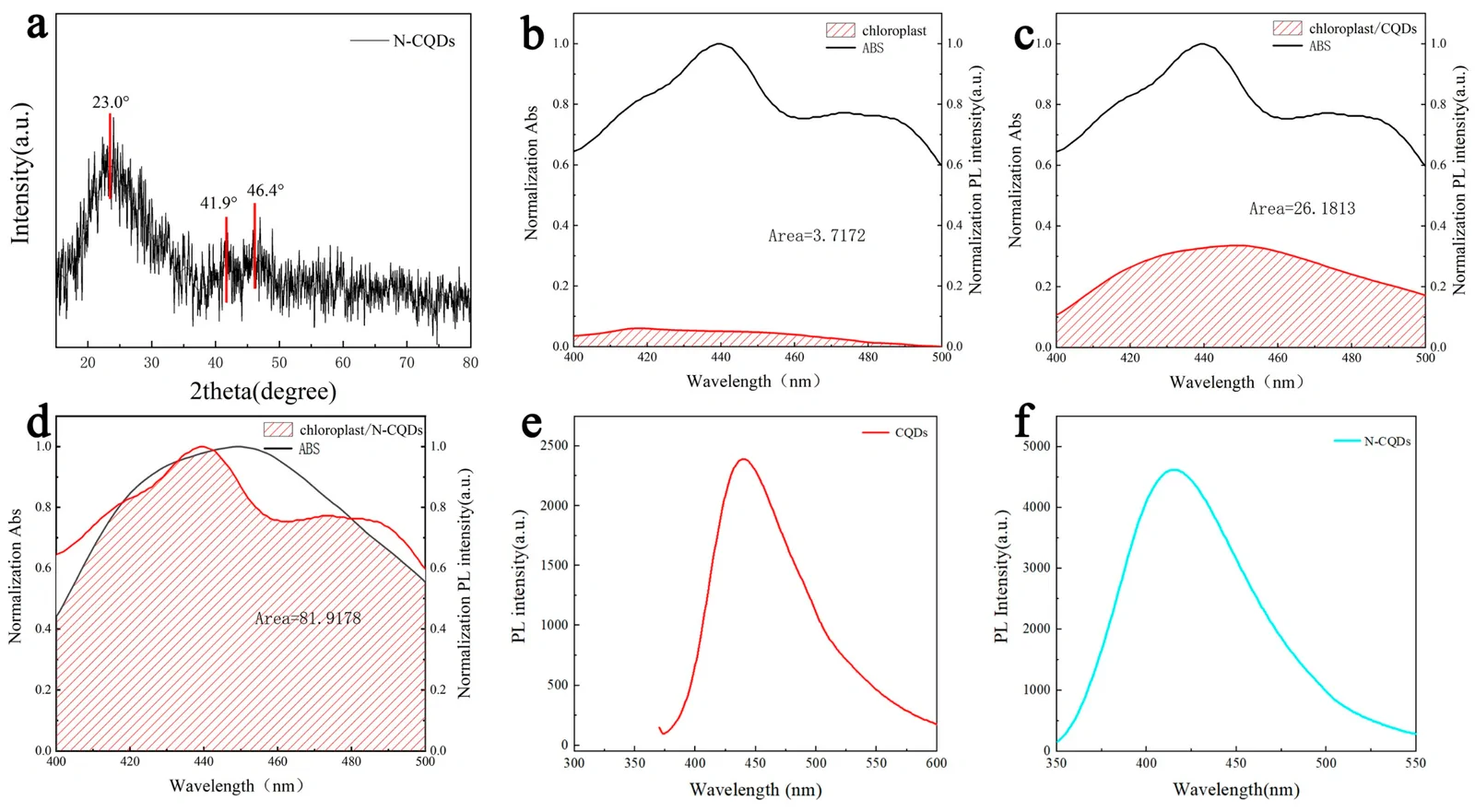
(**a**) XRD spectra of N-CQDs; normalized integral diagram of absorbance and fluorescence intensity of chloroplast-fluorescence spectrum excitation wavelength was 365 nm ((**b**): chloroplast; (**c**): CQDs chloroplast complex; (**d**): N-CQDs chloroplast complex); (**e**) CQDs emission spectra; (**f**) Emission spectra of N-CQDs.

The CQDs and N-CQDs were characterized by energy dispersive spectroscopy (EDS), and the results are shown in Figures S1 and S2. By comparing the element content, it can be seen that the presence of N signal indicates that the nitrogen-doped lignin-based carbon quantum dots prepared by urea composite carbon quantum dots, namely N-CQDs, are successfully prepared. Subsequently, the morphology and microstructure of N-CQDs were characterized by TEM. As shown in Figure S3, N-CQDs showed a spherical monodisperse nanoparticle structure and were well dispersed. After citric acid modification, the ball diameter is small and the particle distribution is uniform. The histogram (Figure S3a) shows that the diameter of these points is evenly distributed between 0.5 and 4.5 nm, with an average size of 2.19 nm and a standard deviation of 0.85 nm. The particle size detection results of N-CQDs are shown in Figure S3a, and the particle size of N-CQDs is between 0.5 and 4.5 nm and shows a normal distribution. It can be seen that the particle size distribution of N-CQDs is uniform, and the formed solution system is relatively stable. At the same time, the illustration in Figure S3b is a high-resolution TEM image, which shows the crystallinity of N-CQDs. It can be clearly observed that the lattice fringe spacing of N-CQDs is 0.21 nm, which is consistent with the diffraction surface of graphite (100) and conforms to the single crystal graphite carbon lattice structure. The XRD pattern of N-CQDs is shown in Figure 3a, and the (100) peak is a more significant peak at 2θ = 41.9°. It is mainly related to the arrangement of aromatic rings in graphite carbon, and the (100) peak is weak in high-quality graphite carbon. The peak at 2θ = 23.0°, corresponding to the (002) peak of graphite carbon, reveals the existence of amorphous carbon structure. The (101) peak of graphite carbon is located at about 2θ = 46.4°, which is consistent with the TEM test results [21]. Combined with infrared spectroscopy, XPS spectroscopy, EDS, TEM and XRD test results, it can be proved that N-CQDs with nano-size and uniform particle size distribution were successfully prepared.

### 2.3. Optical Response and Excited State Dynamic Behavior

Photoluminescence (PL) is one of the unique optical properties of CQDs. In order to study the optical properties of CQDs, the UV-visible absorption spectrum and fluorescence spectrum were measured, and the optical photos were added as shown in Figure 3 and Figure S4.

Figure S4a shows the absorption peak of CQDs in the UV-visible wavelength range. The UV-visible absorption spectrum shows that CQDs have a significant absorption peak at 291 nm, which is mainly attributed to the n-π of C=O bond and the π-π electron transition of the C=C bond [22,23]. Compared with the UV-Vis absorption spectrum Figure S4b of N-CQDs, the obvious absorption peak appears at 350 nm, which is also attributed to the n–π of C=O bond and the π–π electron transition of C=C bond. The conjugated coupling of the functional groups on the surface of CQDs and the carbon core has an important influence on the fluorescence performance of CQDs. At the same time, due to the doping of N element, the UV-visible absorption peak of N-CQDs has a red shift relative to the UV-visible absorption peak of CQDs. This red shift may be due to the reduction in the HOMO-LUMO gap after the formation of graphite nitrogen in the structure after N doping. The deep quantum dynamics quantitative deconstruction was performed by the chloroplast absorbance and fluorescence intensity normalized integral map shown in Figure 3b–d. The blue fluorescence of the CQDs chloroplast complexes and the N-CQDs chloroplast complexes overlaps with the absorption of chloroplasts (Figure 3b–d), which is larger than the area of chloroplasts themselves, indicating that chloroplasts have higher light-harvesting ability after compounding CQDs and N-CQDs. Fluorescent nanomaterials can improve the efficiency of light conversion (LC), and N-CQDs can improve the ability of light conversion, thereby improving the photosynthesis of crops [24].

In order to systematically evaluate the effect of nitrogen doping on the photophysical and chemical properties of carbon dots, this study compared the photoluminescence (PL) spectra of CQDs and N-CQDs (as shown in Figure 3e,f). Under the same excitation conditions, CQDs showed a weak fluorescence emission peak at about 440 nm. In contrast, N-CQDs after nitrogen doping showed significantly enhanced fluorescence emission. The maximum emission peak is at about 415 nm, and the intensity is almost twice that of CQDs. In addition, compared with CQDs, the emission peak of N-CQDs shows a significant blue shift (~25 nm), indicating that nitrogen doping has a significant regulatory effect on the electronic band structure of carbon dots. The significant improvement in the fluorescence performance of N-CQDs can be attributed to the introduction of nitrogen atoms and their surface passivation effect. On the one hand, the electronegativity of nitrogen (3.04) is greater than that of carbon (2.55), and it is rich in lone pair electrons. When nitrogen atoms are doped into the sp^2^ carbon skeleton (such as the formation of pyridine nitrogen or amino groups), they can conjugate with the π-conjugated system of carbon dots, effectively regulate their energy band structure, and introduce new and more efficient radiative recombination active centers. On the other hand, the surface nitrogen-containing functional groups act as excellent surface passivators, which can effectively eliminate the non-radiative traps on the surface of carbon dots and inhibit the non-radiative recombination process, thereby greatly improving the radiative recombination efficiency of excitons. The blue shift in the emission peak is closely related to the fine-tuning of the quantum size effect caused by nitrogen doping and the change in the surface non-uniform local stat [25,26]. In summary, PL spectrum analysis strongly confirmed the significant superiority of the nitrogen doping strategy in optimizing the photoluminescence performance of carbon dots and improving the efficiency of radiation transition, which provided a strong support for the subsequent enhancement of the chloroplast’s capture of sunlight, energy conversion, and transfer efficiency in photosynthesis.

## 3. The Deep Physiological Mechanism of Carbon Quantum Dots Promoting Photosynthetic Carbon Fixation of Biomass Materials

### 3.1. Transport of N-CQDs in Soybean Seedlings and Trans-Barrier Permeability of Vascular Bundles

**Figure 4:**
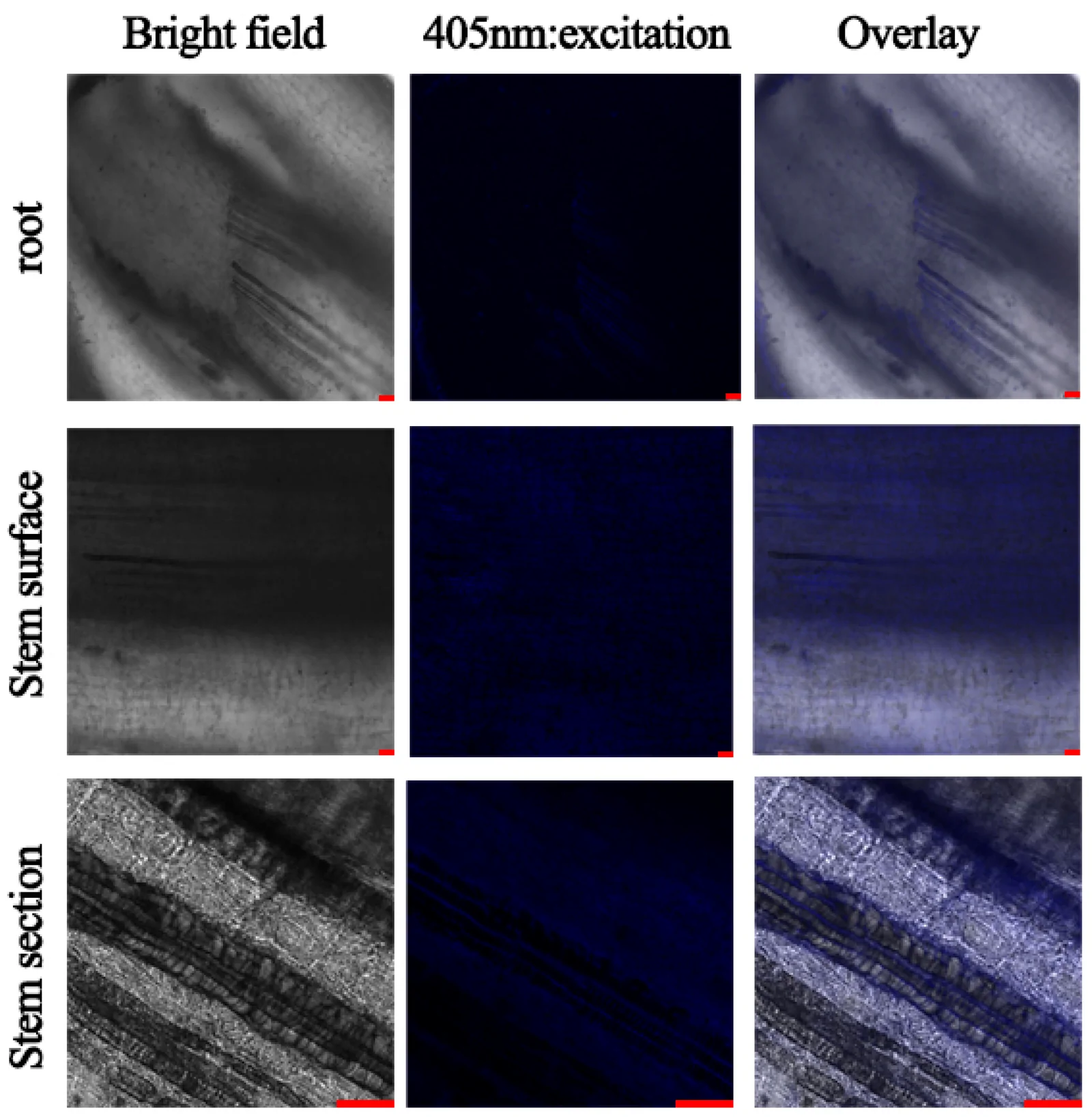
Laser confocal images of soybean sprouts (the red scale bar in the bottom-right corner of each panel represents 50 µm).

N-CQDs have excellent fluorescence properties. Since the volume of N-CQDs is very small, it can easily penetrate plant tissues, pass through barriers, and pass through plant systems. Therefore, an appropriate amount of N-CQDs was sprayed during the growth of soybean seedlings, and the absorption of soybean seedlings was observed by laser confocal scanning microscopy (CLSM) using the blue luminescence emission of N-CQDs. The microscopic test parameters were set to 405 nm excitation to reduce the autofluorescence signal. The soybean seedlings treated with N-CQDs are shown in Figure 4. The fluorescence signal of N-CQDs can be clearly observed. N-CQDs are transferred from the roots to the stems of bean sprouts through the vascular system. This is mainly because the small size and high surface reactivity of N-CQDs can penetrate plant cells or be absorbed by plants, and affect the growth and development of plants. The bright field and luminescence images of the root, stem surface and stem section were observed, and they overlapped well. The fluorescence images of the plants visually showed that the luminescence signal of N-CQDs was mainly in the root hair area, which further indicated that N-CQDs entered the vascular bundle and then transferred upward to the stem through the vascular system, indicating that this is a feasible strategy to enhance photosynthesis in vivo and in vitro [27].

### 3.2. Hill Reaction and Kinetic Behavior of Photosynthetic Electron Transport Chain

The electron transfer rate from PSII to PSI can be measured by the Hill reaction, and its activity can be characterized by the reduction rate of DCPIP. In order to verify whether CQDs and N-CQDs can absorb blue light converted by ultraviolet light for photosynthesis, this experiment discussed the effect of CQDs and N-CQDs on the reduction in DCPIP by chloroplast alone by observing the complexes of CQDs and N-CQDs with chloroplasts. The whole process of biochemical reaction kinetics driven by simulated sunlight is carried out strictly according to the Hill reaction equation shown in Figure S5a.

The reaction kinetics was studied by monitoring the change in absorbance, and the electron reduction rate was measured. The kinetic characteristic curve is shown in Figure S5b. The N-CQDs particles and CQDs particles free in the system had almost no kinetic fluctuation within 5 min, and the reduction in chloroplast DCPIP combined with CQDs and N-CQDs increased significantly, indicating that the electron transport rate on the photosynthetic electron transport chain was accelerated. Therefore, it can be considered that chloroplasts absorb blue light converted by CQDs and N-CQDs to improve photosynthetic activity and promote photosynthesis. Notably, the complex of N-CQDs and chloroplasts showed a higher DCPIP reduction rate, indicating that N-CQDs produced more electrons than CQDs, which may be due to the fact that N doping promoted the separation of photoexcited electron-hole pairs [28].

**Figure 5:**
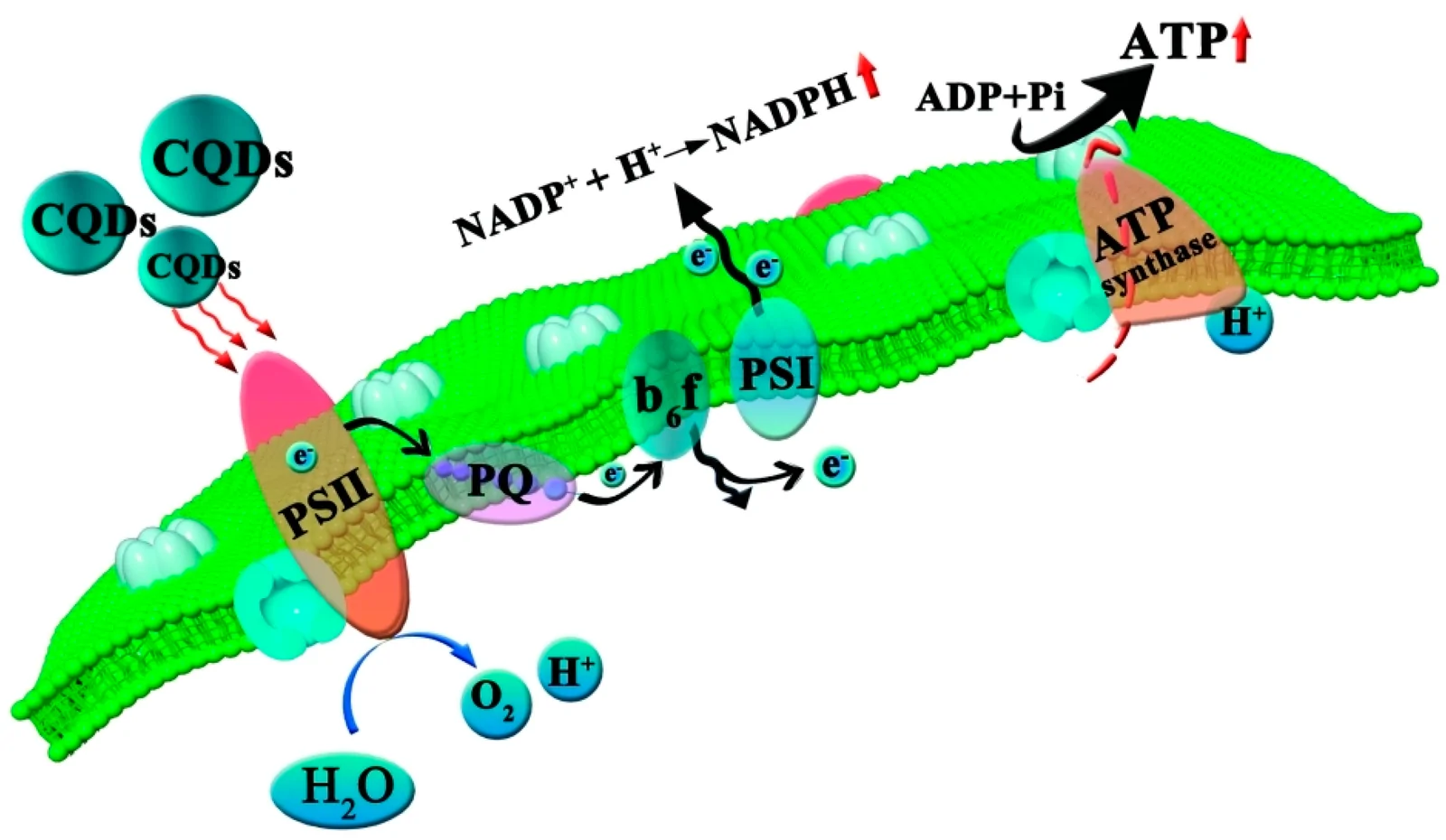
Photosynthetic electron transport (thylakoid metabolism) in plant leaves.

Through this biochemical kinetic test, this study thoroughly clarified the biochemical mechanism panorama of exogenous application of salix powder-based N-CQDs regulators to enhance photosynthesis from the bottom layer (as shown in Figure 5). The electrons extracted from PSII in water are transferred to PSI through the plastid quinone (PQ) pool, cytochrome b6/f complex (Cyt b6/f) and plastocyanin (PC), and finally reduced to NADPH. CQDs have a positive effect on PSII activity and electron transport, which helps to reduce the non-photochemical energy loss of photosynthesis and enhance photochemical energy conversion. The enhancement of photosynthetic activity in soybean plants is closely related to the biosynthesis and metabolism of chlorophyll activated by CQDs and N-CQDs. ATP and NADPH produced by light reaction directly determine the accumulation of plant biomass. In summary, CQDs and N-CQDs improved the photoreaction and carbon dioxide assimilation in photosynthesis by regulating (1) chlorophyll content, (2) light absorption, and (3) photosynthetic electron transport, and ultimately promoted the growth of soybean plants.

## 4. The ‘Low Promotion and High Inhibition’ Rule of Carbon Quantum Dot Regulation on the Evolution of Soybean Cellulose/Lignin Components

### 4.1. Evolution of Soybean Whole Life Cycle Phenotype and Accumulation Kinetics of Non-Structural Carbohydrates

**Figure 6:**
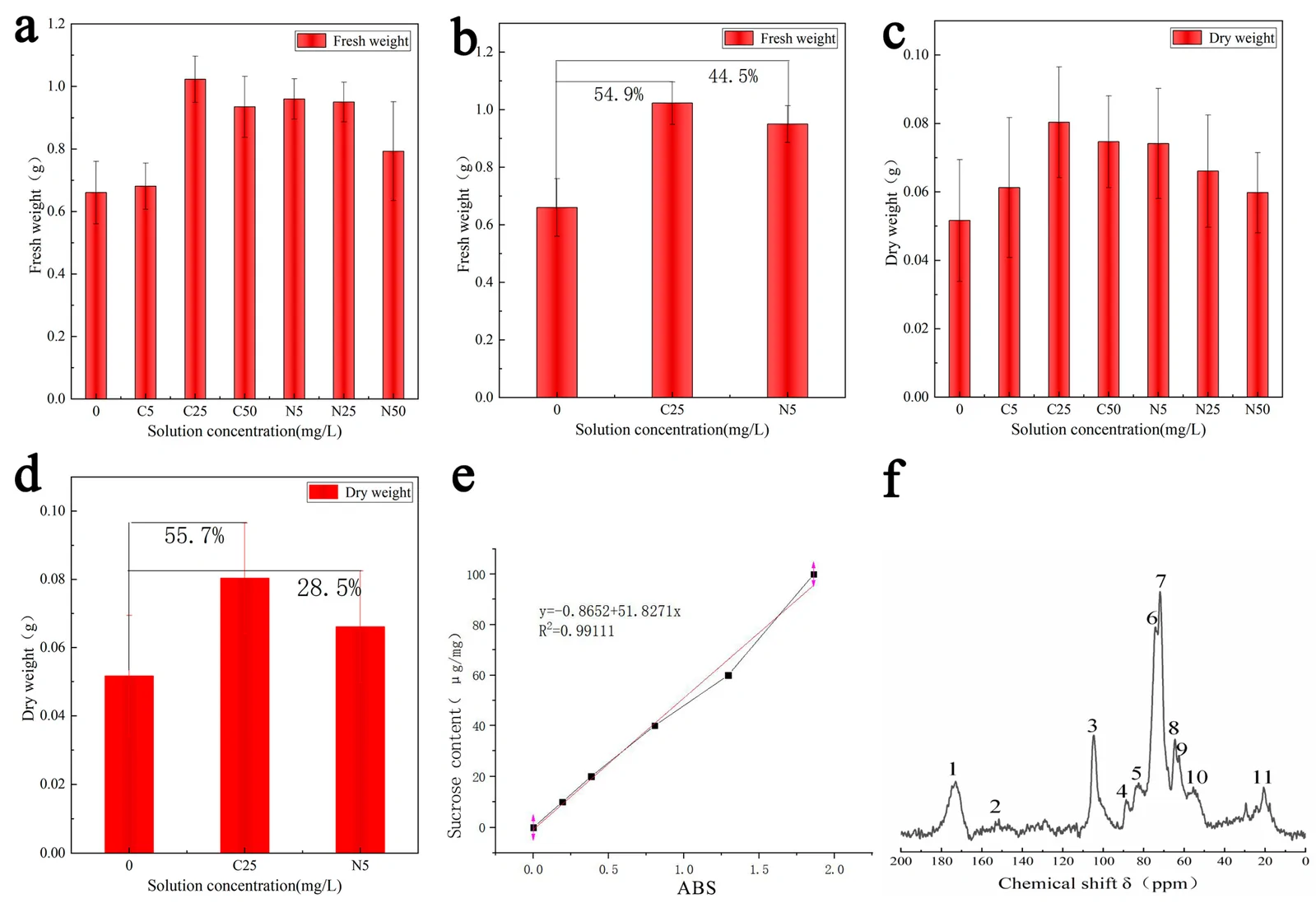
The changes in fresh weight of soybean plants (**a**) The effects of CQDs and N-CQDs on the fresh weight of soybean plants; (**b**) Comparison of N-CQDs and CQDs; the effect of CQDs and N-CQDs on the dry weight of soybean plants; (**c**) the effect of CQDs and N-CQDs on the dry weight of soybean plants; (**d**) Comparison of N-CQDs and CQDs; (**e**) Soluble sugar equation diagram; (**f**) ^13^C NMR spectra of soybean stem.

During the growth of soybean seedlings (Figure 6a,b), the fresh weight showed an increasing trend after spraying with CQDs. Compared with 0.66 g in the natural growth group, the average growth rate was 33.2%, and the highest value was 54.9% when the concentration of CQDs was 25 mg/L. Compared with the control group (0.66 g), the fresh weight after spraying N-CQDs exhibited an average increase of 36.4% across the tested concentrations, reaching a peak increase of 45.4% at 5 mg/L (N5). For dry weight, the N5 treatment yielded a 28.5% increase over the control. At this time, the concentration of N-CQDs was 5 mg/L. After treatment with CQDs and N-CQDs, the fresh weight of soybean seedlings showed an overall growth trend, but in the treatment group, the growth rate showed low promotion and high inhibition with the change in concentration—that is, there was an extreme value. Figure 6c,d represent the dry weight of soybean seedlings measured when they are dried to constant weight. After treatment with CQDs and N-CQDs, the results were similar to the changes in wet weight, showing an increasing trend, and the effect in the group showed low promotion and high inhibition. However, from the perspective of growth rate, the average growth rate and maximum growth rate under the action of CQDs were close to fresh weight, while N-CQDs decreased, which may be due to the fact that N-CQDs promoted the transport of water and volatiles more than CQDs [29].

Sugar is the main product of photosynthesis, which enables plants to accumulate energy and produce organic matter. Soluble sugars can affect gene expression by participating in metabolic activities such as sugar transport and plant growth and development. In this study, the absolute content of soluble sugar in soybean stems was determined strictly according to the standard linear regression equation of soluble sugar concentration characteristics made by the standard colorimetric method (as shown in Figure 6e, y = −0.8652 + 51.8271x, R^2^ = 0.99111).

**Figure 7:**
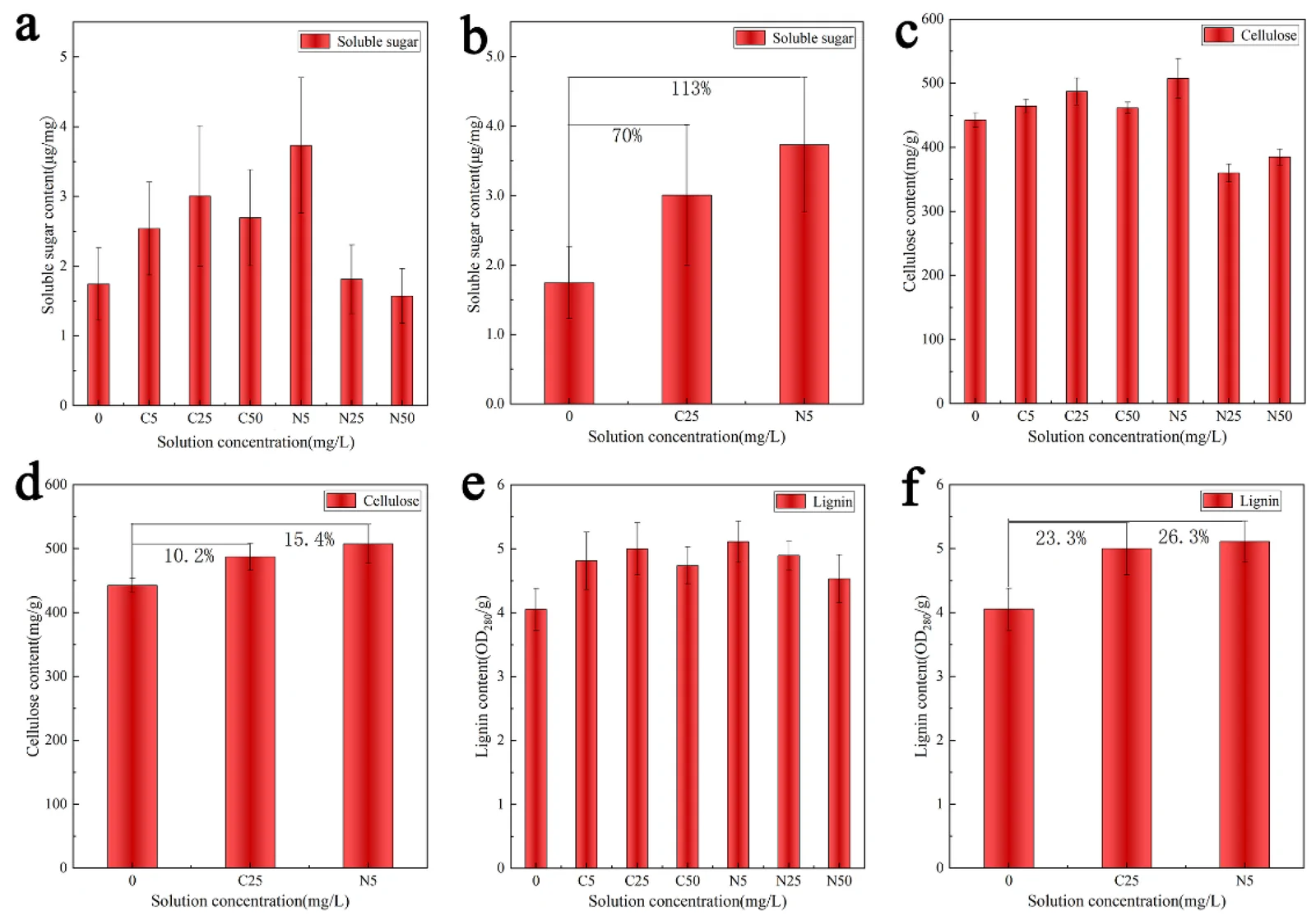
Changes in soluble sugar in soybean plants ((**a**): effects of CQDs and N-CQDs on soluble sugar in soybean plants; (**b**): comparison of N-CQDs and CQDs); (**c**) the effect of CQDs and N-CQDs on the cellulose content of soybean stem; ((**d**): comparison of N-CQDs and CQDs); (**e**) the effect of CQDs and N-CQDs on the lignin content of soybean stem; ((**f**): comparison of N-CQDs and CQDs).

As shown in Figure 7a,b, compared with the control group, the soluble sugar of spraying CQDs at 5 mg/L, 25 mg/L and 50 mg/L increased by 43%, 70% and 53%, respectively. Obviously, the soluble sugar content reached the highest at 25 mg/L, and showed a slight downward trend at 50 mg/L. It is indicated that under the appropriate CQDs concentration, it can effectively promote the accumulation of sugar and the growth of soybean seedlings. When spraying N-CQDs at 5 mg/L, 25 mg/L and 50 mg/L, the soluble sugar increased by 113% and 5% at low concentrations of 5 mg/L and 25 mg/L, and the soluble sugar content at 50 mg/L N-CQDs concentration was 9% lower than that of the control group. The soluble sugar content reached the highest at 5 mg/L, decreased significantly at 25 mg/L, and inhibited at 50 mg/L. The results showed that the appropriate concentration of CQDs and N-CQDs could promote the soluble sugar production of soybean seedlings, but N-CQDs with better fluorescence performance had a certain inhibitory effect at high concentration [30,31].

### 4.2. Solid-State NMR Structure Identification and Quantitative Deconstruction of Stem Structural Carbohydrates (Cellulose/Lignin)

Nuclear magnetic resonance spectroscopy (NMR) is one of the effective methods to study the structure of lignin. Figure 6f shows the solid ^13^C NMR spectrum of soybean stem powder. In the ^13^C NMR spectrum, the signals related to the three main components of the lignocellulose structure, lignin, cellulose and hemicellulose, were expressed. The specific chemical shift attribution is shown in Table S2.

After completing the above phase identification, the absolute mass fraction evolution of the backbone core polymer-cellulose in each group of soybean straw was accurately measured. The results are shown in Figure 7c,d. Compared with the control group, the cellulose content of spraying CQDs at 5 mg/L, 25 mg/L and 50 mg/L increased by 4.5%, 10.2% and 3.8%, respectively. The cellulose content of CQDs treatment group reached the highest value of 487 mg/g at 25 mg/L, and showed a downward trend at 50 mg/L. The effect of low promotion and high inhibition was more obvious under the action of N-CQDs. After spraying N-CQDs at 5 mg/L, 25 mg/L and 50 mg/L, cellulose produced the best promotion effect at a low concentration of 5 mg/L, increasing by 15.4%, while the cellulose content at 25 mg/L and 50 mg/L N-CQDs concentration showed an absolute inhibition effect compared with the control group, which decreased by 18.8% and 17.7%, respectively.

The lignin content in the stems of fresh plants was further determined by the acetylated lignin method, as shown in Figure 7e,f. Compared with the control group, the cultivation of CQDs and N-CQDs showed a promoting effect. The lignin content of spraying CQDs at 5 mg/L, 25 mg/L and 50 mg/L increased by 18.5%, 23.3% and 16.7%, respectively, and the promotion effect was the best at the concentration of 25 mg/L. The lignin content of spraying N-CQDs at 5 mg/L, 25 mg/L and 50 mg/L increased by 26.3%, 21.2% and 11.5%, respectively, and the promotion effect reached the highest value at the concentration of 5 mg/L. Combined with all the above tests, spraying different concentrations of CQDs and N-CQDs would affect the fresh weight, dry weight, soluble sugar, cellulose and lignin content of soybean straw. The results showed that low concentration promoted high concentration inhibition, and the growth and development promotion effect of soybean plants at low concentration of 5 mg/L was N-CQDs > CQDs.

### 4.3. Spectra-Specific Micro Staining and Micro-Distribution Deconstruction of Straw Tissue

In this study, the lignin deposition at 3 cm from the root of soybean plants was detected by saffron solid green staining with histochemical microscopy, and the distribution of lignin deposition in the stems of soybean plants was explored.

**Figure 8:**
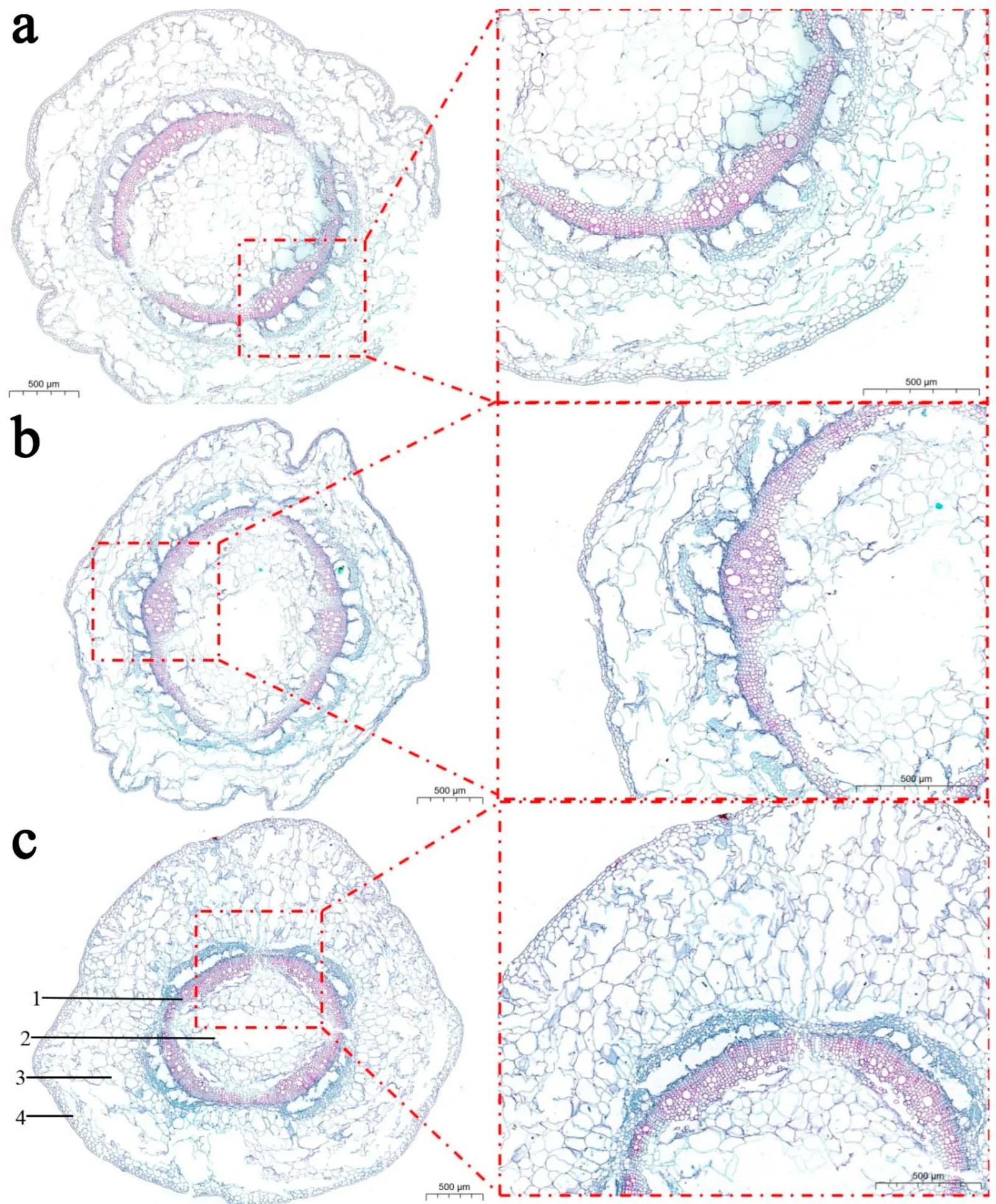
Saffron solid green staining of soybean stem (scale = 500 μm, 1. xylem, 2. medullary cavity, 3. cortex, 4. epidermis), a, b, c represent different positions of stem.

The staining results are shown in Figure 8. The control group (Figure 8a), CQDs treatment group (Figure 8b) and N-CQDs treatment group (Figure 8c) were compared. The results showed that the cross-sectional staining area density of the CQDs treatment group and the N-CQDs treatment group was higher, indicating that lignin accumulated more. Lignin specific histochemical staining showed that compared with the control group, the lignification of the stem vascular system of CQDs and N-CQDs plants was enhanced, and the lignin deposition was increased. At the same time, the xylem fibroblasts of the control group were arranged more closely, and the cell wall thickness of the xylem tissue of the treatment group was thicker than that of the control group, but the difference was not significant. The staining results showed that spraying CQDs or N-CQDs on soybean plants could effectively promote the accumulation of lignin in plant cell wall.

## 5. The Mapping and Structure–Activity Relationship of Macroscopic Mechanical Strength Enhancement of Biomass Materials

### Macroscopic Shear Resistance (Toughness) and Micromechanical Tensile Stress–Strain Behavior Evolution of Soybean Straw

**Figure 9:**
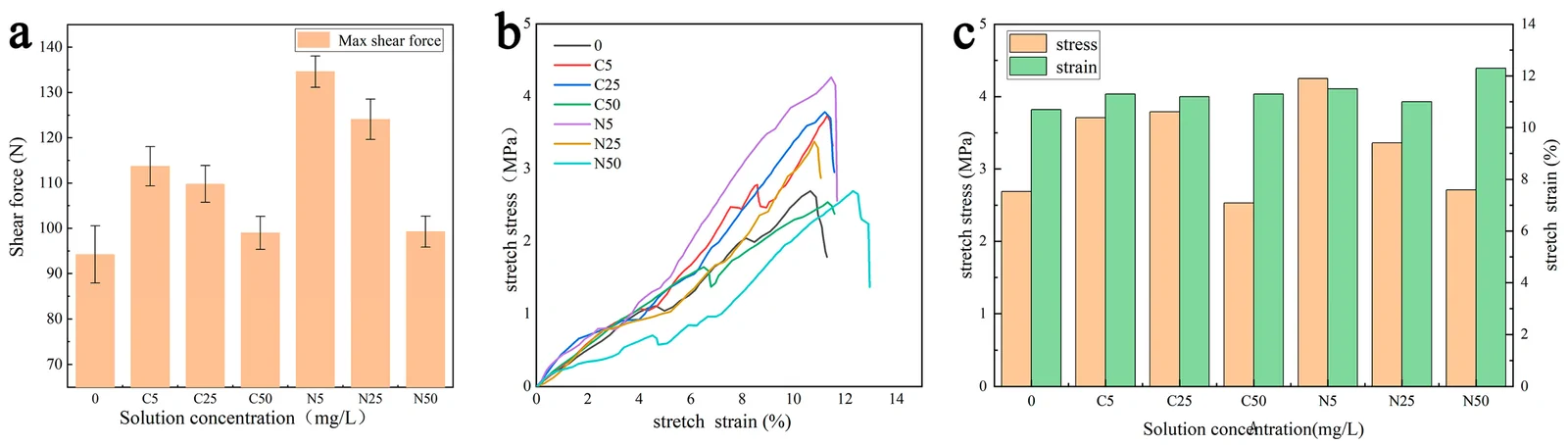
Mechanical properties of soybean stems. (**a**) Shear mechanical properties (maximum shear force, N); (**b**) tensile stress–strain curves across different treatment concentrations (distinguished by color-coded profiles); (**c**) maximum tensile stress (MPa, orange bars, left axis) and fracture strain (%, green bars, right axis) under various CQD and N-CQD concentrations.

The shear force of soybean stem was measured by texture analyzer, and the effects of CQDs and N-CQDs on promoting photosynthesis and increasing lignin accumulation on plant stem toughness were analyzed. The shear toughness of soybean stems is shown in Figure 9a. Taking the concentration of CQDs and N-CQDs treatment solution as a variable, the shear force of soybean stems increases with the increase in cellulose and lignin content. Under the action of CQDs, the cellulose content and lignin content showed an increasing trend, so the shear toughness of soybean stems also increased compared with the control group. Under the action of N-CQDs, when the concentration of N-CQDs was 5 mg/L, both cellulose and lignin reached the maximum accumulation, so the shear force that could be tolerated also reached the maximum value of 134.76 N. After the concentration of N-CQDs treatment solution increased, the shear force of soybean stem decreased sharply.

Figure 9b is the tensile stress–strain diagram of soybean stems. The effects of cellulose and lignin accumulation on the toughness of soybean stems under the action of CQDs and N-CQDs were investigated. Combined with the above changes in the accumulation of structural carbohydrates in soybean stems, the tensile stress and strain of soybean stems correspond to the accumulation of cellulose and lignin. The accumulation of cellulose and lignin under the action of CQDs increased compared with the control group, so the mechanical tensile properties of soybean stem also increased compared with the control group. Under the action of N-CQDs, when the concentration of N-CQDs was 5 mg/L, both cellulose and lignin reached the maximum accumulation, which produced the best mechanical tensile properties, and the tensile force was 4.25 MPa. After the concentration of N-CQDs treatment solution increased, the decrease in cellulose content led to a sharp decline in the mechanical properties of soybean stems. The lignin in the soybean stem mainly provides rigidity. The greater the rigidity, the greater the brittleness, and the easier it is to break. Cellulose plays a role in skeleton toughness, so the shear force and tensile properties of soybean stem are mainly affected by cellulose accumulation. The tensile stress and tensile strain diagram of soybean stem (Figure 9c) showed that the tensile stress increased by 40.9% and the tensile displacement increased by 5.3% on average under the action of CQDs, and the tensile stress increased by 58% and the tensile displacement increased by 25.2% on average under the action of N-CQDs. When the concentration of N-CQDs treatment solution was 50 mg/L, the decrease in cellulose content led to the minimum tensile stress of soybean stem of 2.21 MPa, which was consistent with the above test results of cellulose and lignin content in soybean stem.

Combined with the mechanical test results, the mechanical properties of soybean stalks exhibited a close correlation with the secondary accumulation of structural carbohydrates. Lignin acts as a rigid three-dimensional cross-linked network that determines the initial matrix stiffness, whereas the linear polysaccharide chains of cellulose microfibrils confer skeleton toughness. Consequently, the maximum shear load-bearing limit (134.76 N) and tensile fracture strength (4.25 MPa) are predominantly dominated by the coordinated accumulation of cellulose and lignin under optimal N-CQD regulation (5 mg/L).

Therefore, this work has completely established the following fiber-level structure–activity mapping relationship: 1. Lignin, as a grid adhesive network structure, determines the initial rigid performance of the matrix material. 2. Cellulose provides skeleton toughness due to its linear polymer polysaccharide long-chain microfibrillar grid structure, which directly dominates and maps the maximum shear load bearing limit and fracture tensile strength of macro-stems.

## 6. Conclusions

In this study, highly luminescent and crystalline nitrogen-doped carbon quantum dots (N-CQDs) with a uniform spherical particle size of 0.5–4.5 nm (average size of 2.19 nm) and distinct single-crystal graphite (100) lattice fringes of 0.21 nm were successfully synthesized from natural biomass Salix powder using urea and citric acid as a nitrogen source and activator, respectively. The synthesized quantum dots exhibited efficient ultraviolet absorption and down-conversion blue light emission, which, upon entering the plant vascular system as traced by laser confocal scanning microscopy, significantly improved the light-harvesting capacity of chloroplasts, accelerated the thylakoid photosynthetic electron transport chain, and promoted the Hill reaction rate. Under whole-plant regulation, the accumulation of non-structural and structural carbohydrates strictly followed a “low promotion and high inhibition” dosage rule: compared with the untreated control, the optimal CQD treatment (25 mg/L) increased fresh and dry weights by 54.9% and 55.7%, respectively, while the optimal N-CQD treatment (5 mg/L) enhanced seedling fresh weight by 45.4%, dry weight by 28.5%, soluble sugar by 113%, and stem cellulose and lignin mass fractions by 15.4% and 26.3%, whereas higher concentrations (50 mg/L) exhibited evident inhibition. Crucially, a fiber-level structure–activity mapping relationship between microscopic polysaccharide evolution and macroscopic physical mechanics was established, wherein the coordinated accumulation of cellulose and lignin under 5 mg/L N-CQD treatment effectively enhanced stem toughness, maximizing the shear force to 134.76 N and peak tensile strength to 4.25 MPa. This closed-loop framework of “nanomodulator synthesis-photosynthetic carbon assimilation-cell wall microcomponent evolution-macroscopic mechanical enhancement” provides a viable biotechnology strategy for the targeted performance regulation of agroforestry biomass functional materials.

## Data Availability

The data used to support the findings of this study are available from the corresponding author upon request.

## Supplementary Materials

The following supporting information can be downloaded at: https://www.mdpi.com/article/10.3390/polym18202538/s1, Table S1: Sucrose buffer configuration; Table S2: ^13^C nuclear magnetic resonance chemical shift attribution table of soybean stem; Figure S1: EDS energy spectrum of CQDs; Figure S2: EDS energy spectrum of N-CQDs; Figure S3: TEM image of N-CQDs; Figure S4: Optical properties of CQDs and N-CQDs ((a): UV and fluorescence spectra of CQDs; (b): UV and fluorescence spectra of N-CQDs); Figure S5: CQDs, N-CQDs, chloroplast, CQDs chloroplast complex, N-CQDs chloroplast complex photosynthetic activity diagram ((a): Hill reaction equation; (b): DCPIP reduction rate diagram).
