# Supplementary material for: Wood-Based Carbon Quantum Dots Regulate Photosynthetic Carbon Fixation and Enhance Mechanical Properties of Soybean Straw
Source: Polymers (Basel). 2026 Oct 16;18(20):2538. doi: 10.3390/polym18202538 (PMC13708822; doi:10.3390/polym18202538)
Supplement: Supplementary file 1 [file polymers-18-02538-s001.zip › polymers-4584404-supplementary.pdf]

Wood-Based Carbon Quantum Dots Regulate Photosynthetic Carbon Fixation and Enhance Mechanical Properties of Soybean Straw

Yujia Fu[a,1], Fengqi Qiu[a,1], Sihan Tu[a], Zhenhua Xue[a,b,c,*]

[a] College of Material Science and Art Design, Inner Mongolia Agricultural University, Hohhot, 010018, China

[b] Inner Mongolia Key Laboratory for Sand Shrubs Fibrosis and Energy Development and Utilization.

[c] National Forestry Grassland Engineering Technology Research Center for Efficient Development and Utilization of Sandy Shrubs.

[1] These authors contributed equally to this work and should be considered co-first authors.

* E-mail addresses: x_zhenhua@126.com.

S1. Specific details of the precursor preparation process

1.1. Centrifugation Parameters: After hydrothermal reaction, the crude reaction mixture (30 mL) was transferred to 50 mL polypropylene centrifuge tubes and centrifuged at 8000 r/min for 10 min at 25°C to precipitate large particulates. The supernatant was then passed through a 0.22 μm polyethersulfone (PES) syringe filter.

1.2. Membrane Dialysis Parameters:

Membrane Specification: Regenerated cellulose dialysis tubing with a Molecular Weight Cut-Off (MWCO) of 1000 Da (purchased from Shanghai Yuanye Bio-Technology Co., Ltd., China).

Volume and Replacement: 20 mL of the filtered raw solution was loaded into the dialysis bag and dialyzed against 2000 mL of deionized water in a glass beaker. The dialysis medium (2000 mL deionized water) was stirred continuously and replaced with fresh deionized water every 6 h over the 48 h purification period (a total of 8 water changes).

1.3. Freeze-Drying Parameters:

Freezing: 15 mL of the dialyzed, purified CQD/N-CQD solution was transferred to a pre-cooled vessel and pre-frozen at -80°C for 12 h in an ultra-low temperature freezer.

Lyophilization: The pre-frozen sample was then placed in a freeze-dryer (SCIENTZ-18N, Ningbo Scientz Biotechnology Co., Ltd., China) and lyophilized under a vacuum pressure of <10 Pa at a cold trap temperature of -55°C for 48 h until completely dehydrated solid powders were obtained.

S2. Preparation of 1 chloroplasts and carbon quantum dots and N-doped carbon quantum complexes

Chloroplast extraction: Firstly, 30mL (pH = 7.3) sucrose buffer was prepared according to the formula shown in Table 6. The prepared sucrose buffer was placed in the refrigerator and stored at 4°C for use. An appropriate amount of spinach leaves rich in chloroplasts were weighed and fully ground in a mortar containing 30 mL sucrose buffer. The chloroplast crude extract was obtained by filtration, and the chloroplast precipitate was obtained after three times of centrifugation in the dark (the first centrifugation, the chloroplast crude extract was centrifuged at 1000 rpm for 3 min, and the bottom precipitate was discarded to retain the supernatant. After the second centrifugation, the retained supernatant was centrifuged at 3000 rpm for 5 min and the

precipitate and supernatant were collected respectively. The third centrifugation. The supernatant after the second centrifugation was centrifuged at 3000 rpm for 5 min and the precipitate was collected). Finally, the collected sediment was re-dispersed in sucrose buffer to obtain chloroplast suspension. All the above operations need to be carried out under dark conditions of 0 ~ 4°C.

Determination of chloroplast suspension concentration: 1mL undiluted chloroplast suspension was mixed with 49 mL acetone, and the absorbance of the mixed solution at 652 nm was measured to calculate the concentration of chloroplast suspension (Formula S1). After repeated experiments, it was determined that the concentration of chloroplast suspension diluted with sucrose buffer was 5 mg / L for Hill reaction.

$$C = OD_{652} \times 1000/34.5 \tag{S1}$$

In the formula: C is the concentration of chloroplast (element), unit is mg/L; OD_{652} is the absorbance value of the suspension at a wavelength of 652 nm.

Table S1. Sucrose buffer configuration.

Reagent	Concentration
KCl	0.01 mol/L
Na_2HPO_4	0.03 mol/L
Sucrose	0.4 mol/L
KH_2PO_4	0.02 mol/L

Preparation of chloroplasts and carbon quantum dots and N-doped carbon quantum complexes: CQDs sucrose buffer, N-CQDs sucrose buffer and chloroplast suspension with a concentration of 5mg/L were prepared. The CQDs chloroplast complex suspension was prepared by mixing CQDs sucrose buffer with chloroplast suspension at a volume ratio of 1:1, and the N-CQDs chloroplast complex suspension was prepared by the same method.

S3. Effects of N-CQDs and CQDs on photosynthetic activity of chloroplasts

The photosynthetic activity of chloroplasts is generally reflected by the Hill reaction, which measures the electron transport rate of photosystem II during photosynthesis. The reaction process is represented by the absorbance change of the dye 2,6-Di-chloropheno-lindopheno (DCPIP), that is, the reduction rate. Firstly, the five groups of suspensions were fully mixed for 5 min, and then the appropriate amount of 60 μmol/L DCPIP was added for 1 min. The suspension was irradiated by a full-spectrum lamp to simulate photosynthesis. The experimental groups were placed under the light, and the absorbance value (λ = 600 nm) was measured in parallel before

and after illumination every minute. The whole process of simulated photosynthesis test was 6 min.

S4. Test method

S4.1. Soybean seed treatment

The soybean seeds with full particles, uniform size, no incomplete and moth-eaten were selected. The surface impurities and dust were cleaned by deionized water, and then 0.1 % KMnO4 solution was used to clean and disinfect for 10 min. After washing with deionized water for three times, the soybean seeds were soaked for 24 h, and then evenly spread in a 4-layer petri dish with paper towels on the bottom layer, and cultured in shading until the bean sprouts grew to 1-2 cm. The germination process was kept away from light, and 50 mL distilled water was used to wash once every 12 h.

S4.2. Soybean plant cultivation

The experiment was carried out in the material test room of Inner Mongolia agricultural college students. The germinated soybean seeds were moved into plastic baskets, and soybean seedlings were cultivated with pure water (0 mg/L control group), CQDs and N-CQDs aqueous solution test groups with concentrations of 5 mg/L, 25 mg/L and 50 mg/L, respectively. Daily light 16 hours dark 8 hours. The vertical distance of the lamp to soybean seedlings was 0.5 m, and each sample was treated three times. After 10 days of cultivation, the growth and development indexes were measured.

S4.3. Determination of fresh weight and dry weight of soybean stem

The fresh weight of soybean plants was measured by analytical balance. Three strains of soybean plants in the same period cultured with control group 0 mg/L, CQDs 5 mg/L, 25 mg/L, 50 mg/L and N-CQD 5 mg/L, 25 mg/L, 50 mg/L were selected, and their fresh weight was recorded after wiping the surface. Then, the plants were separately wrapped in kraft paper, killed at 105 °C for 30 min in the oven, and baked at 75 °C until constant weight, and their dry weight was measured.

S4.4. Determination of soybean stem lignin

The lignin content was determined by ultraviolet spectrophotometer.

(1) Test principle: The phenolic hydroxyl group in lignin is acetylated to form acetyl lignin. The product has a characteristic absorption peak at 280 nm, and the content of lignin can be quantitatively detected by the change of absorbance value.

(2) Grinding of soybean stem: The main stems of soybean plants at different growth stages were taken, and the total mass of each stem segment was weighed. Then 0.1g fresh sample was weighed and placed in a mortar (if the mass was more than the required requirement, it should be evenly reduced from top to bottom, if less than the required mass, the sample was re-selected). Appropriate amount of quartz sand and 95 % ethanol were added and ground to homogenate.

(3) Soybean stem slurry drying: The slurry was transferred to a 2 mL centrifuge tube, centrifuged at 4500 r/min for 10 min, poured out the supernatant, collected the precipitate, and added an appropriate amount of 95 % ethanol to the EP tube. Wash the precipitate three times, and then rinse with a mixture of ethanol: n-hexane = 1:1 (v/v) three times, collect the precipitate, and place the precipitate in a fume cupboard until it is completely dried.

(4) Lignin acetylation: 0.2 mL of 25% bromoacetyl (glacial acetic acid configuration) solution was added to the EP tube containing dry precipitate to dissolve, cover the EP tube cover, place it in a water bath at 70 °C for 30 min, and mix it upside down every 10 min to ensure that the drug is in full contact with the precipitate, and 0.16 mL 2 mol / L NaOH was added to terminate the reaction.

(5) Determination of absorbance: 2 mL glacial acetic acid and 0.04 mL 7.5 mol/L hydroxylamine hydrochloride were added immediately after the termination of the reaction, and mixed evenly. After centrifugation at 4500 r / min for 5 min, 0.1 mL of the supernatant was taken and diluted with 3.0 mL of glacial acetic acid. The absorbance value was measured at OD280 nm, and the lignin content (in fresh weight) was expressed as OD280/g.

S4.5. Determination of cellulose content in stem of soybean

(1) Determination principle: Cellulose reacts with sulfuric acid and anthrone. When the sample is mixed with concentrated sulfuric acid at high temperature, sulfuric acid will decompose cellulose into glucose monomer and oxidize it into carboxylic acid. Anthrone is added to the reaction system as an indicator and reacts with carboxylic acid to form colored products. Therefore, the content of cellulose is determined by measuring the absorbance of colored products.

(2) Removal of fat and starch: The sample was dried to constant weight at 80 °C, crushed, and passed through an 80-mesh sieve. About 0.1 g (denoted as W) was weighed in a 2 mL centrifuge tube, 80 % ethanol was added to remove sugar, and acetone was added to remove fat. Then 1 mL dimethyl sulfoxide solution (remove

starch) was added to soak for 15 h, and centrifuged at 4000 rpm at 25 °C for 10 min. The supernatant was discarded and the precipitate was dried. The weight was the dry weight of the cell wall.

(3) Determination of cellulose content: About 5 mg of dried cell wall was weighed, and 0.5 mL of deionized water was added to fully homogenize. The homogenate was transferred to an EP tube, diluted to 0.5 mL with deionized water, placed in an ice water bath, slowly added 0.75 mL of concentrated sulfuric acid, mixed well, and stood in an ice water bath for 30 min. Centrifugation was performed at 8000 rpm (4 °C) for 10 min. The supernatant was diluted 20 times with distilled water and then analyzed. Take 300 uL test solution, add 70 uL anthrone ethyl acetate and 630 uL concentrated sulfuric acid, water bath at 95 °C for 10 min, read the absorbance value A620 at 620 nm, and calculate the cellulose content according to Formula S2.

$$C = 4.76\times(A_{620} + 0.0043) / W \qquad (S2)$$

In the formula: C is the cellulose content, the unit is mg/g; the absorbance value of A620 solution at wavelength of 620 nm; w is the sample weight, and the unit is g.

S4.6. Determination of Soluble Sugar in Soybean Stems

(1) Test principle: Sugar can be dehydrated to form furfural or hydroxymethylfurfural under the action of concentrated sulfuric acid. The generated furfural or hydroxymethylfurfural can react with anthrone to form blue-green furfural derivatives. Within a certain range, the color depth is proportional to the content of sugar, so it can be used for quantitative determination of sugar. The carbohydrate content measured by the anthrone method is actually the total amount of soluble carbohydrates in the solution. The absorption peak of the colored substance produced by the reaction of saccharides and anthrone in the visible light region is 630 nm, so the color comparison is carried out at this wavelength.

(2) Preparation of standard curve: add standard sucrose solution, and then add 0.5 mL of ethyl acetate reagent and 5 mL of concentrated sulfuric acid to the test tube in order. After sufficient oscillation, the test tube is immediately put into a boiling water bath, and the test tube is accurately kept for 1 min one by one. After taking it out, it is naturally cooled to room temperature. The optical density is measured at 630 nm wavelength with blank as control. The standard curve is drawn with the optical density as the ordinate and the sugar content as the abscissa, and the standard linear equation is obtained.

(3) Test of soluble sugar: The stem of fresh soybean seedlings was extracted, the surface dirt was wiped off, cut into pieces and mixed evenly, and 0.1 g was weighed, a total of 3 portions. They were placed in 3 scale test tubes, added with 5-10 mL distilled water, sealed with plastic film, extracted in boiling water for 30 min and repeatedly extracted twice. The extract was filtered into a 25 mL volumetric flask, and the test tube and residual liquid were repeatedly rinsed to constant volume. Colorimetric determination 0.5 mL of sample extract was taken into a 20 mL scale test tube (repeated twice), and 1.5 mL of distilled water was added. The following steps were the same as the standard curve determination, and the optical density of the sample at 630 nm wavelength was determined.

S4.7. Determination of cellulose lignin distribution in soybean stem

The plant tissues were stained by saffron solid green staining test to observe the degree of lignification. Safranine is an alkaline dye that can show the lignification, corkification and cutinization of vascular plants. Fast green is an acid dye, which is a kind of dye containing pulpy cellulose cell tissue. The catheter can be stained with safranin, and the sieve tube can be stained with fast green. The soybean stems were selected from the root 2 cm away from the root of the stem for hydration, safranin staining 1 h water washing after fixation of green staining 30 s after dehydration section under the microscope.

S4.8. Determination of chemical structure of cellulose lignin in soybean stem

Bruker-500,400MHz standard cavity, conventional cross polarization (CP) and devil 's rotation (MAS) were used for testing.

Table S2. ^{13}C nuclear magnetic resonance chemical shift attribution table of soybean stem.

Peak no	δ (ppm)	Chemical shift assignment
1	173.45	Cγ, acetyl carbon-COOR of ferulic acid and p-coumaric acid
2	152.52	C3/C5 of non-phenolic S-type lignin
3	104.61	C1 of cellulose and hemicellulose
4	88.35	Cα in lignin β-5 structure
5	82.67	Cα in β-β structure of lignin and Cβ in β-O-4 structure of G-type lignin.
6	74.05	C1, C2 of cellulose and hemicellulose and Cα in β-O-4 structure
7	71.72	C2, C3, C5 of cellulose and hemicellulose, Cα in β-O-4 structure.
8	64.39	C6 in cellulose and hemicellulose, Cγ in β-5, β-1 and β-O-4 in lignin.
9	62.70	Cγ in lignin β-O-4 structure
10	54.73	Methoxyl group
11	20.79	Acetylated methyl

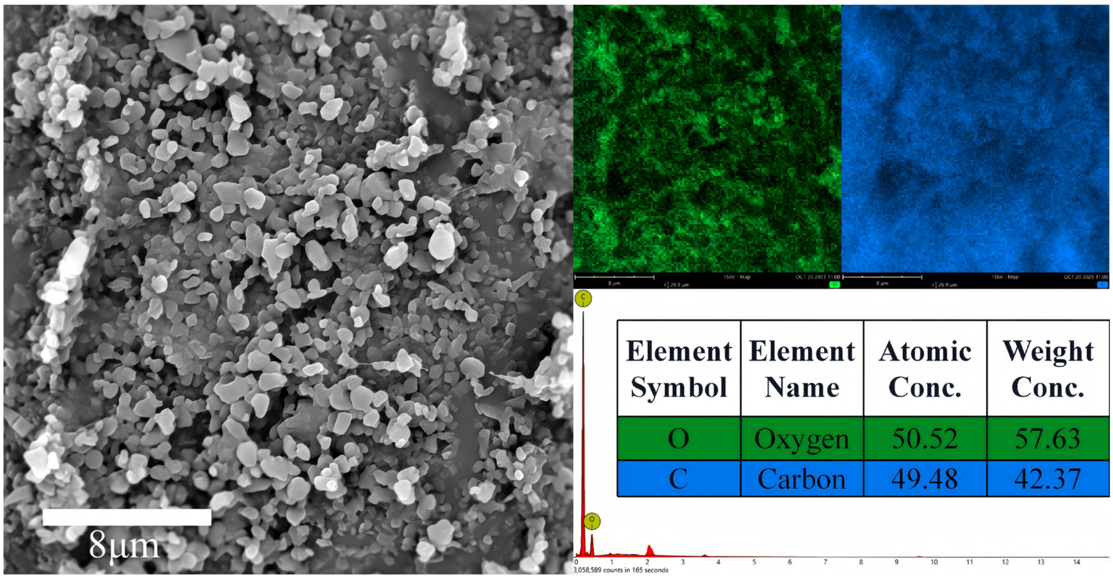

Figure S1. EDS energy spectrum of CQDs

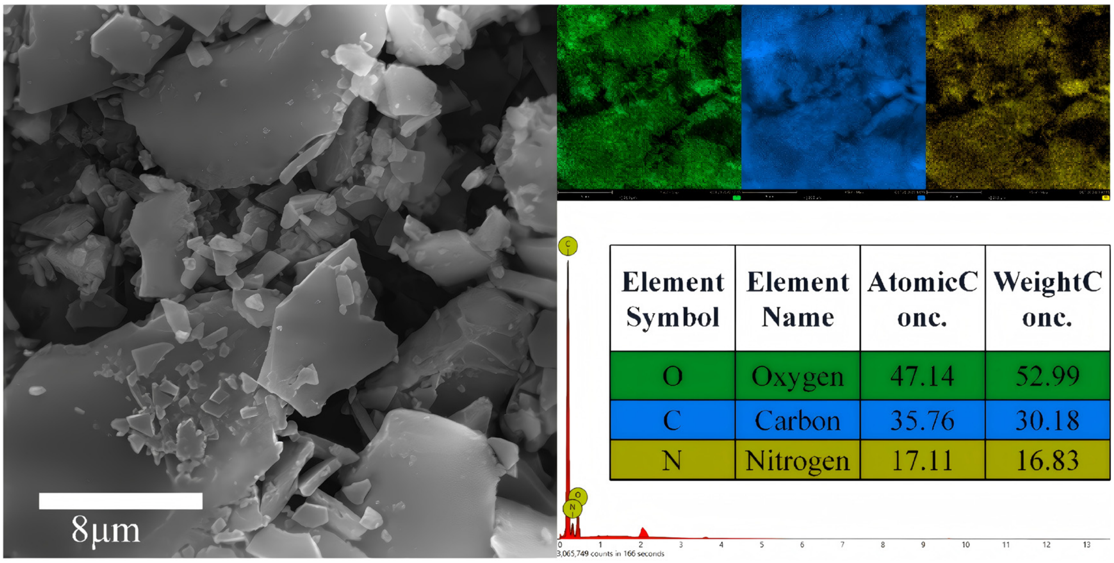

Figure S2. EDS energy spectrum of N-CQDs

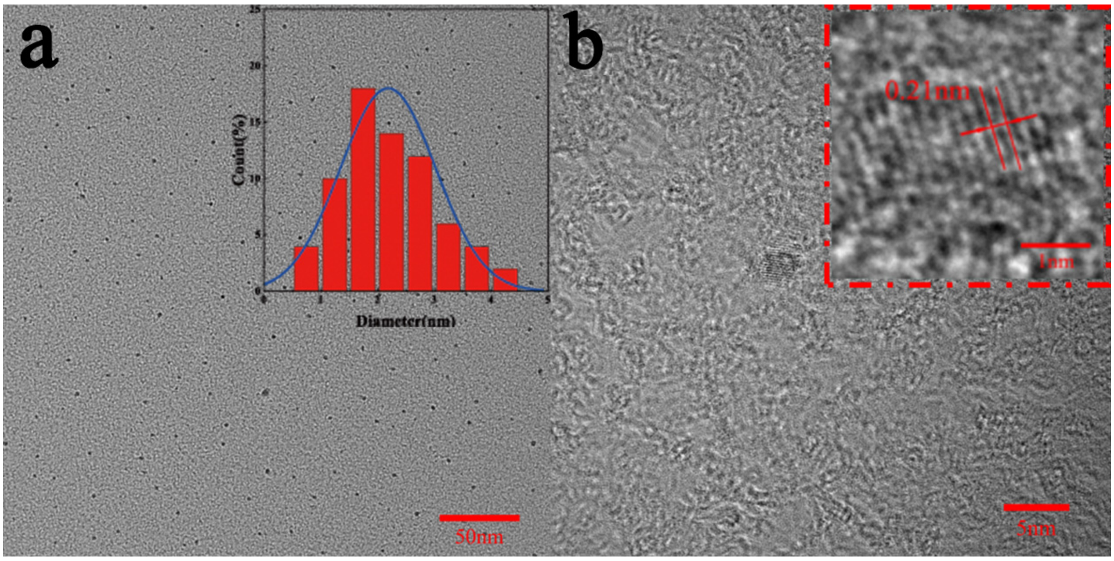

Figure S3. TEM image of N-CQDs

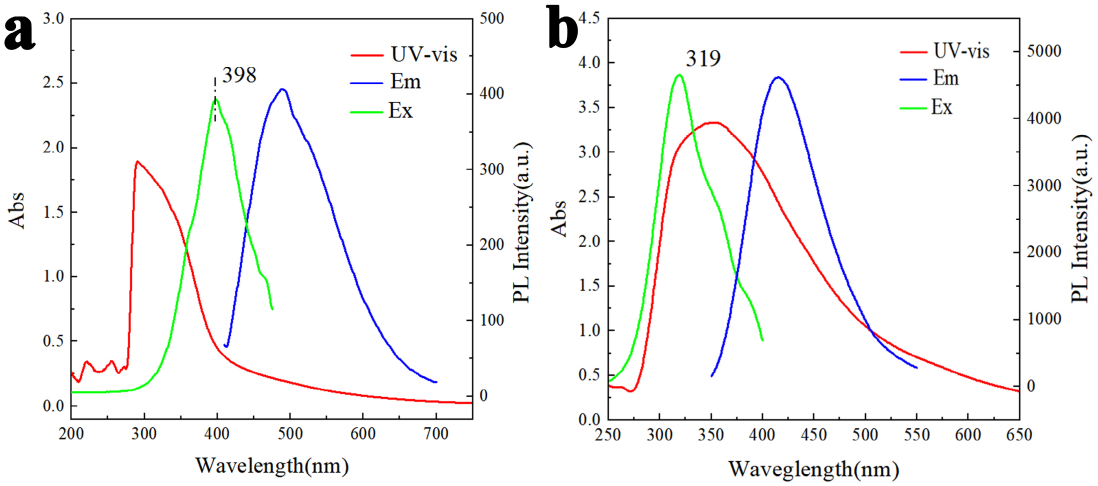

Figure S4. Optical properties of CQDs and N-CQDs (a: UV and fluorescence spectra of CQDs; b: UV and fluorescence spectra of N-CQDs).

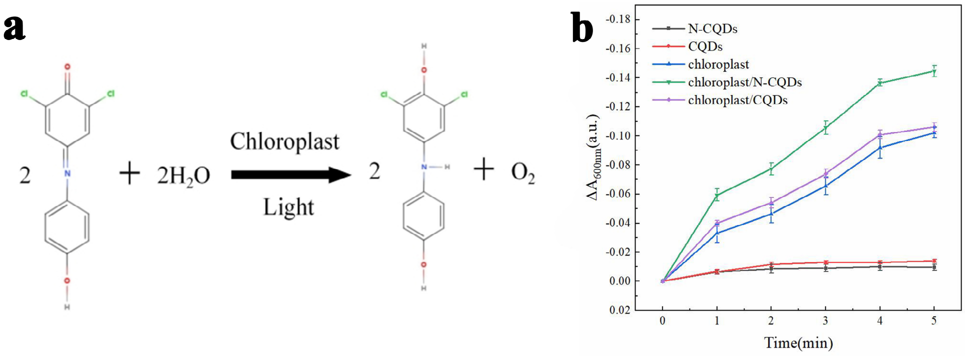

Figure S5. CQDs, N-CQDs, chloroplast, CQDs chloroplast complex, N-CQDs chloroplast complex photosynthetic activity diagram (a: Hill reaction equation; b: DCPIP reduction rate diagram)
